# In‐depth assessment of snacking behaviour in unmarried adolescent girls 16–19 years of age living in urban centres of Java, Indonesia

**DOI:** 10.1111/mcn.12833

**Published:** 2019-08-06

**Authors:** Lauren S. Blum, Ayu Mellisa, Eny Kurnia Sari, Isma Novitasari Yusadiredja, Marti van Liere, Susan Shulman, Doddy Izwardy, Ravi Menon, Alison Tumilowicz

**Affiliations:** ^1^ Global Alliance for Improved Nutrition (GAIN) Jakarta Indonesia; ^2^ PT Kadence International Jakarta Indonesia; ^3^ Global Alliance for Improved Nutrition (GAIN) Geneva Switzerland; ^4^ Directorate of Public Health Ministry of Health Republic of Indonesia Jakarta Indonesia

**Keywords:** adolescent girls, eating behaviours, Indonesia, qualitative research, snack food consumption

## Abstract

Adolescence is a critical period characterized by physical, social, and developmental changes that impact on health and eating behaviour. Indonesia is experiencing dramatic economic and infrastructural changes, causing greater access to the global food industry and media. This transition is influencing food intake trends, leading to new nutritional challenges in adolescent girls. Qualitative research was conducted between November 2016 and January 2017 in five urban sites in Java, Indonesia, to examine individual, social, environmental, and macrosystem factors affecting snacking behaviours in unmarried adolescent girls 16–19 years of age. Methods entailed 30 freelisting exercises, nine key informant interviews, and 16 in‐depth interviews. Freelisting results identified over 200 snack foods, with the most salient processed convenience foods such as chips and cookies. Respondents typically snacked multiple times daily. Widespread availability of affordable and “tasty” snacks makes snack foods appealing meal substitutes. Snacks provide a distraction to boredom and loneliness and an enhancement to social gatherings. Girls exhibited limited understanding or concern about potential negative effects of snacking. Parents facilitate acquisition of nutrient‐poor snacks, whereas friends exert pressure for routine consumption of snack foods. Social media infiltrated with promotions of eateries and snack foods is likely contributing to the preponderance of snack food consumption. Routine consumption of snack foods high in sugar, salt, and fat and skipping meals will likely have long‐term consequences on the nutritional status and health of Indonesian adolescent girls. Findings underline the urgent need to develop contextually relevant, targeted behavioural change strategies to modify the potentially harmful eating and activity patterns of adolescent girls identified in this study and to curb the trajectory of overweight in urban Indonesia.

**Key messages**

- Adolescent girls snack multiple times daily on foods high in sugar, salt, and fat, leading to meal skipping.
- The motivation to snack is primarily driven by the exotic flavours and alluring tastes offered by cheap, unhealthy snacks saturating Javanese cities.
- Snacking serves as an antidote to boredom and loneliness and is considered central to socializing.
- Adolescent girls have limited understanding of snack food contents or concerns about the negative effects of snacking.
- Unless trends of excessive snacking and meal skipping are addressed, there will likely be a further rise in overweight and obesity among female adolescents and adults.

## 1 INTRODUCTION

Adolescence is a transitional period in the life cycle characterized by physical, social, and developmental changes that influence health and eating behaviour. Starting at the onset of puberty and continuing to young adulthood, the World Health Organization (WHO) defines adolescence as between 10 and 19 years of age, with the period commonly divided into early (10–14 years) and late (15–19 years) adolescence (Black et al., 2013; G. C. Patton et al., 2018). During this period, rapid growth and dramatic physical changes create increased demand for nutrients and energy as simultaneously, adolescents undergo many psychosocial changes, such as seeking independence and self‐identity and growing concerns about body image, all of which can impact on food selection and nutritional intake (Das et al., 2017; Story, Neumark‐Sztainer, & French, 2002). Further, the nutritional status of females before and during pregnancy is critical for the health and survival of the mother and her baby (Black et al., 2013; Han, Mulla, Beyene, Liao, & McDonald, 2011). Research has consistently shown that adolescents frequently have less than optimal eating habits, increasing the risk for nutritional problems, including undernutrition, overweight and obesity, iron deficiency anaemia, and micronutrient deficiencies (Akseer, Al‐Gashm, Mehta, Mokdad, & Bhutta, 2017). The combination of these factors makes adolescence a nutritionally vulnerable period (WHO, 2005).

Understanding nutritional problems in adolescents is important in lower income and middle‐income countries where there has been a rise in noncommunicable diseases while malnutrition persists (Popkin & Gordon‐Larsen, 2004; Rivera, Pedraza, Martorell, & Gil, 2014). Escalating incomes and urbanization, as well as improved communication and the expansion of the global processed food industry, have increased access to packaged energy‐dense, nutrient‐poor convenience foods and fast food outlets (Popkin, 2017). Economic and social changes have led to modifications in diet and physical activity and are linked to a nutritional transition involving a rapid rise in the incidence of overweight and obesity (Popkin, 1999; Popkin & Gordon‐Larsen, 2004). In many lower income and middle‐income countries, marriage and first birth still occur at a young age when girls are maturing, leading to increased nutritional demands (Gibbs, Wendt, Peters, & Hogue, 2012; WHO, 2014). Moreover, adolescent behaviours involving food choices, eating frequency, and the social context of food consumption are found to be predictive of adult eating practices (Craigie, Lake, Kelly, Adamson, & Mathers, 2011; Larson, Neumark‐Sztainer, Hannan, & Story, 2007; Merten, Williams, & Shriver, 2009; Pedersen, Holstein, Flachs, & Rasmussen, 2013).

Indonesia, the fourth most populated country in the world, is experiencing rapid socio‐economic changes affecting food accessibility and intake, which in turn impacts on nutritional status, including that of adolescent girls (United Nations, 2017). Overweight and obesity has increased over the past two decades across all age groups (Rachmi, Li, & Alison Baur, 2017). Prevalence is highest in female adolescents and adults, with a third of women over 18 years of age overweight or obese, whereas close to a 10th of girls in the 16–18 age group are reported to be overweight or obese (National Institute of Health Research and Development, 2013). At the same time, national survey data show that approximately half of nonpregnant girls aged 15–19 are undernourished (as indicated by mid‐upper arm circumference measurements <23.5 cm) and a fourth of females between 15 and 24 years suffer from anaemia (Health Research and Development Agency, 2013). These studies highlight a complex double burden of undernutrition and overweight present in Indonesian adolescent girls (Roemling & Qaim, 2013).

A limited number of sociobehavioural studies have been conducted to understand the causes of overweight in Indonesian adolescents. One study demonstrated that perceptions that fast food is modern and practical, limited physical activities, and high intake of soft drinks and processed foods contributed to obesity, with adolescents from higher income families three times more likely to be obese (Collins, Pakiz, & Rock, 2008). Another study showed that decreased physical activity and high consumption of modern foods were linked to higher body mass index (Roemling & Qaim, 2012). Although overweight and obesity in Indonesia has been associated with higher socio‐economic status and urban residency, recent studies showing an increase in prevalence of households with a double burden of malnutrition suggest that a shift may be occurring with overweight affecting a broader socio‐economic spectrum (Rachmi, Li, & Alison Baur, 2018). Snacking is reported to be pervasive in Indonesia (Global Alliance for Improved Nutrition, 2014), as well as among adolescent girls (Kadence International, 2014). Consumption of fried foods including oily and fried snacks more than four times per week has been associated with overweight among children and adolescents (Prihatini & Jahari, 2007).

A conceptual model designed to understand and explain adolescent food choices and consumption behaviours identifies broad levels of influences including individual (psychosocial, biological, knowledge, and lifestyle), environmental (family, friends, schools, worksites, fast food outlets, and convenience stores), and macrosystems (socio‐political changes, food availability, food production and distribution, and media and advertising) (Story et al., 2002). Although most studies focus on single‐factor determinants of food intake and nutritional status at the household and individual levels, this ecological model facilitates an exploration of multiple levels of influence, including often neglected broader community and societal determinants, and enhances an understanding of their relationships (Madjdian, Azupogo, Osendarp, Bras, & Brouwer, 2018; Story et al., 2002). This conceptual framework can be useful when examining the multifaceted, dynamic factors affecting food consumption, and the way they interact in influencing adolescent eating behaviours.

Given the spectrum of nutritional challenges faced by Indonesian adolescent girls, social science research is needed to understand better factors affecting eating behaviours to inform intervention strategies. Using the model, we carried out qualitative research to examine the ways in which individual motivations, social and environmental factors, and media and advertising influence eating practices, with a focus on snacking behaviours of girls aged 16–19 in urban Java, Indonesia. The findings provide context‐specific information for the future development of policies and programmes designed to promote healthy eating behaviour in adolescent girls.

## 2 RESEARCH METHODS

### 2.1 Study setting and population

The research was carried out in five urban centres in Java, namely, Central Jakarta, East Jakarta, Surabaya, Yogyakarta, and Malang, over a 5‐week period between November 2016 and January 2017. Selection of research sites was designed to focus on hubs across the island that ranged in geographic locations, population size, economics, and cultural backgrounds. The study concentrated on unmarried adolescents aged 16–19 years, an age group in Indonesia shown to be susceptible to overweight and obesity, representing three categories of girls who typically have contrasting schedules and lifestyles including high school students, college students, and girls working full‐time.

### 2.2 Study design, sampling, and methods of measurement

We employed complementary qualitative data collection methods using structured, semistructured, and open‐ended techniques to assess snacking behaviours as described below.

- Freelisting is a structured exercise used to generate a preliminary inventory of the cultural domain of snack foods and associated terminology (Pelto & Pelto, 1978). We carried out two freelisting exercises, asking respondents to list (a) commonly consumed snack foods and (b) motivations for consumption of snack foods. Exercises were administered to girls living in the five research sites. Using purposive sampling to select unmarried female adolescents aged 16–19 years representing the three study categories of girls, high school and college girls were identified in education institutions and malls known as “hang out” places, and employed girls were approached in shopping and trade centres where they frequently work. The initial aim was to carry out freelisting with 30 adolescent respondents.
- In‐depth interviews with adolescent girls were designed to explore individual motivations and environmental influences guiding eating behaviours of adolescent girls, especially snacking, including how they choose snacks, where and when they obtain and eat snacks, and the social context in which snacks are consumed. Eligible high school and college girls were identified on school campuses, and employed girls were approached for interviews in shopping centres where they worked. Inclusion criteria established prior to the study were as follows: high school students aged 16–17 years enrolled in a private, public, or vocational school and living with their family; college students 18–19 years of age enrolled in a college or university full‐time and living either with their family or in a dorm; and workers 18–19 years, employed full‐time, and living with their family or on their own. Girls who had participated in the freelisting exercises were not eligible to partake in the in‐depth interviews.
  A recruitment questionnaire that had been previously tested for validity and reliability and employed by the data collectors was used to identify girls living in families from a range of socio‐economic backgrounds. Potential respondents were asked a series of questions related to the household economic status such as the materials used for construction and size of their home, family assets, type of transport family members use, and source of drinking water. Each response had a precoded score, with scores tallied once the questionnaire was completed. Selection criteria were used to ensure that girls represented a range of socio‐economic status with the overall target to include eight high school students, four college students, and four working adolescent girls in the study.
- Key informant interviews were conducted to examine motivations for and patterns of eating and snacking behaviours and recommendations for behavioural change strategies from a diverse spectrum of informal and formal experts on adolescence. Respondents were selected purposively based on their background and expertise as it relates to the study topic and included mothers, food sellers, journalists, and health experts, with most key informants located in Jakarta.
- Informal observations of food venues were carried out in the study sites. Researchers were asked to observe, record, and describe the eating environment respondents were exposed to routinely.

### 2.3 Data collection procedures

Study instruments were administered by seven experienced qualitative researchers assigned to different city sites. Prior to data collection, a 3‐day training was held to introduce the study protocol, methods, and ethical procedures and to test and modify the instruments. All interviews were administered in bahasa Indonesia, the national language of Indonesia. Key informant and in‐depth interviews were audio recorded; interviewers also took handwritten notes of information that could provide additional insights into the data. Researchers were in regular communication throughout the data collection period, either through a cloud account or through group calls to share preliminary results. An iterative process involving the review of initial findings and additional questioning continued until data saturation was reached. A debriefing meeting involving study investigators was convened at the completion of data collection.

### 2.4 Data analysis

Freelisting exercises were analysed on Anthropac 4.983 software. Salience, which refers to the perceived importance, was derived using a salience index (Smith's *S*) defined as follows: *S* = ((*L* − *Rj*+1)/*L*)/*N*, where *L* is the length of each list, *Rj* is the rank of item *J* in the list, and *N* is the number of lists in the sample (Borgatti, 1999). Items with a higher salience score were considered to have greater significance in the local framework of snack foods (Bernard, 1988; Romney & d'Andrade, 1964; Weller, 1984). The audio‐recorded interviews were translated into English and transcribed in a Microsoft Word document; after reviewing the transcripts, a coding system was developed for the key informant and in‐depth interviews. Coding categories were derived from the initial research themes, as well as from key concepts that emerged during data collection. Coding of the interview transcripts was done in ATLAS.ti, a text‐organizing software. Content analysis was used to identify trends of concepts in and across individual codes. The combination of data and methodological triangulation allowed us to analyse data across the different research methods and across and between respondents (Bernard, 2017; M. Q. Patton, 2015).

### 2.5 Ethics

The study protocol was reviewed and ethical approval granted by the institutional review board at the Atmajaya University, Jakarta, Indonesia. Signed informed consent was obtained from all the freelisting, key informant, and in‐depth interview respondents.

## 3 RESULTS

### 3.1 Background information

Freelisting exercises were administered to 30 girls across the five research sites. Nine key informants were interviewed, including two mothers and two food vendors plus one nutritionist, magazine editor, psychologist, beauty blogger, and food blogger. In‐depth interviews were carried out with eight high school, four college, and four working respondents located in the five sites. Half of the college and working respondents lived apart from their parents with college respondents living in school dorms inhabited only by college students and working respondents residing in boarding houses with kitchen facilities. Employment of working respondents included administrative assistant, cleaner, cashier, and salesperson. Table 1 presents background information of the in‐depth interview respondents.

**Table 1:** Background information of in‐depth interview respondents

Type of respondent/variable	High school (*N* = 8)	College (*N* = 4)	Working (*N* = 4)
Residence			
Central Jakarta	2	0	1
East Jakarta	2	1	0
Malang	1	1	1
Yogyakarta	2	1	1
Surabaya	1	1	1
Age (years)			
16–17	8	0	0
18–19	0	4	4
Religion			
Muslim	3	4	4
Christian	5	0	0
Living arrangements			
With family	8	2	2
Dorm or boarding house	0	2	2
Parents provide regular pocket money	8	4	0
Own smartphone	8	4	4

### 3.2 Freelisting

Freelisting generated 213 different snack foods. Typical of a cultural domain, the procedures elicited a core set of more significant snack items and a wide range of less important foods, with the 25 most salient items listed in Table 2. Core snacks were predominantly modern foods such as chips, cookies, puffed snacks, French fries, and ice cream, whereas traditional foods such as dumplings and *pentol* (skewered meatballs) were considered of less importance. The most salient motivation for consumption of snack foods was taste, followed by easy to get, filling, cheap, and meal substitute, with the designation “healthy” mentioned by only one respondent.

**Table 2:** Most salient snack foods identified through freelisting exercises

Item no.	Local name	English description	Type of snack	Frequency	Average rank	Smith's saliency
1	Keripik kentang	Potato chips	Modern	21	8.8	.522
2	Gorengan	Fried snacks	Traditional	24	12.3	.463
3	Biskuit	Cookies	Modern	27	15.1	.435
4	Kentang goreng	French fries	Modern	23	15.0	.394
5	Es krim	Ice cream	Modern	19	13.4	.331
6	Keripik singkong	Cassava chips	Modern	15	12.2	.299
7	Chiki	Puffed snacks	Modern	16	14.6	.298
8	Siomay	Steamed fish dumplings with vegetables and peanut sauce	Traditional	14	14.1	.272
9	Cilok	Boiled cassava flour made into balls and topped with peanut sauce	Traditional	12	10.1	.249
10	Makaroni	Fried macaroni fried with spicy or salty seasoning	Modern	10	10.6	.247
11	Roti	Bread	Modern	19	18.1	.247
12	Cokelat	Chocolate	Modern	12	13.7	.228
13	Cireng	Fried foods made from cassava as the main ingredient	Traditional	11	12.5	.213
14	Batagor	Fried fish dumplings served with peanut sauce	Traditional	11	14.0	.208
15	Sosis	Sausage	Modern	13	16.1	.187
16	Permen	Candy	Modern	9	16.7	.154
17	Pentol	Skewered meatballs	Traditional	6	7.5	.154
18	Nuget	Nuggets	Modern	10	14.3	.151
19	Teh (botol)	Tea (bottled)	Modern	7	10.9	.138
20	Mie instan	Instant noodles	Modern	6	13.5	.111
21	Lumpia	Spring rolls	Traditional	6	14.0	.105
22	Donat	Donuts	Modern	11	23.6	.104
23	Jamur crispy	Crispy fried mushrooms	Modern	8	21.6	.100
24	Risoles	Small croquette baked or deep fried	Traditional	9	23.8	.100
25	Otak‐otak	Grilled fish cake mixed with tapioca starch and spices	Traditional	8	18.5	.099

### 3.3 Key informant and in‐depth interviews

#### 3.3.1 Individual motivations

##### Food meanings

Adolescents consistently described *makanan* (meals) as heavy, filling foods that stave off hunger over extended periods. These foods are eaten at specific times of the day, including breakfast, lunch, and dinner, and often composed of big portions. The central meal ingredient is rice, the staple and ultimate filling food, with many respondents confirming that without rice, it is not a meal. Other meal foods characterized as having filling properties included noodle‐based dishes, meatballs, meat or chicken, soups, and “Western” foods such as hamburgers, hot dogs, fried chicken, and pizza. When these Western foods are sold in *restoran cepat saji* (fast food chains), they are labelled as junk foods or *makanan cepat saji*, which were described as containing preservatives, processed and stored over a long time, prepared quickly, unhealthy, and delicious. The same foods sold by street vendors or in *warungs* (family‐owned restaurants or food shops) are not considered “junk” because they take time to prepare, highlighting that fast processing signifies junk food. Fast foods were defined as packaged meal items, such as instant noodles, and high in preservatives.

*Cemilan* (snack foods) are described as light and nonfilling and consumed in small portions. *Jajan*, a verb that respondents signified involves buying small foods or snacks, was labelled a common activity among adolescent girls. Snacks are eaten to replace or in‐between meals, with a few respondents calling them *makanan penutup* (literally dessert foods) consumed directly after meals.

Many girl respondents differentiated between modern and traditional snacks, with modern snacks described as packaged, containing preservatives, and branded by a company; traditional snacks are typically not packaged, made with local ingredients, and sold to eat immediately.

##### Lifestyles and eating behaviours

High school students and employed girls had busy, structured schedules, often leaving home at 6 a.m. and returning after 5 p.m., with high school students studying in the evening. College girls have less regimented timetables, with long breaks between classes. Leisure time at home is spent watching television, talking to family members, browsing the Internet, or watching movies online. Socializing involves hanging out with friends in shopping malls and eateries or, for college students, in dorm rooms. Respondents living in boarding houses reported to often be lonely. Most girls play sports infrequently or not at all, suggesting that it is not a priority or they are lazy. Key informants confirmed that adolescent girls admit to being lazy and consider exercising difficult.

Our respondents purchased snacks routinely to consume directly or, especially college students and employed girls, store in the bedroom or kitchen. Snacks are commonly brought in convenience stores, which offer packaged, unperishable foods. Because they do not have a refrigerator, boarders purchase snacks with long shelf life. Some girls buy large quantities monthly in minimarkets or supermarkets, which offer a broad range of items, piquing their curiosity to experiment with unfamiliar snacks. Snacks kept in their living quarters included cookies, chips, candy or chocolate, ice cream, and chocolate cereal, with some stressing the need to have snacks nearby.

Breakfast is often consumed by girls living with parents, whereas boarders frequently skip breakfast. Most girls living at home carried lunch to school, whereas other respondents purchased either a meal or snacks at lunchtime. High schoolers reported eating dinner, whereas college and working respondents often substitute dinner with snacks. A working respondent (R13) living with her parents in Central Jakarta said,

After work, if I don't go out with friends, I stay at home praying, I rarely eat dinner, but snack. Except when I am really hungry, I eat rice. Usually at home I eat snacks; in the fridge I have stocks of wafers, biscuit, chips, ice cream. The first is potato chips and the second is ice cream, those are must‐have snacks.

Skipping meals was more frequent among girls living in boarding houses or dorms, who typically reported not to follow a meal schedule and to more often eat snacks as meal substitutes, especially during breakfast or dinner. This college student (R10) in Malang stated,

Usually my schedule starts at 8 am, sometimes I'm too lazy to go out to buy breakfast, so I eat chips.

Most girls admitted to eating vegetables rarely and only at their parent's home, with many acknowledging a dislike for vegetables. When consumed, vegetables are often in soups or mixed with rice and in small quantities. Several high schoolers reported eating fruits multiple times weekly. Some preferred fruit juice, which is combined with milk and sugar. Respondents did not purchase fruits, and therefore, those living apart from their family did not eat fruit, claiming that fruit is costly, rots quickly if not refrigerated, and can only be obtained in supermarkets.

Our respondents never or rarely cooked, indicating that their mothers are responsible for meals. Despite availability of kitchens in boarding houses, those renting rooms stressed the convenience of buying ready‐to‐eat food. This respondent working in Yogyakarta (R15) said,

There is a kitchen for cooking, but it is too much of a hassle because I need to wake up early in the morning. So I take away the food. I've never thought about that (cooking), I prefer to buy.

The majority of girls reported snacking more than twice daily, indicating that snacking had increased over the past two years and preferring snacks to meals. Reasons for increased snacking cited by high schoolers included the abundance of snack vendors compared with junior high school and that socializing has become more important. College girls pointed to their irregular schedule, increasing opportunities to explore and socialize with friends in cafes or shopping malls, often while snacking. Contrary to high school, college lecturers often permit eating in class. Employed girls indicated greater autonomy and purchasing power. High school and employed girls reported routine snacking when they return home from school or work, and all types of respondents reported to snack at night while studying, watching television, or browsing the Internet often in their bedroom. High school and college girls indicated that snacking is essential when studying in groups.

Many acknowledged less than ideal food consumption behaviours, with some attributing unhealthy eating and the failure to exercise to hectic schedules. Boarders, in particular, highlighted difficulties in maintaining a regular eating schedule. Others reported that dietary regimens only become important at an older age, rationalizing that unhealthy foods are often delicious and difficult to resist. A high school respondent (R7) from Central Jakarta said, “If I like the food, I just eat it, no matter what.”

##### Selection, preferences, and roles of snack foods

Taste was the most important attribute motivating snack food selection across all types of respondents, and in fact, Indonesian snacks come in an array of diverse flavours. Researchers counted over 100 varieties of puffed snacks sold in large plastic bags in one shop. Girls reported that bread, a common snack, is offered with a variety of fillings and toppings such as chocolate, cheese, blueberry, green tea, and banana. Price was the second consideration when choosing snacks. Another perceived advantage was that, as snacks are less filling, they can be eaten multiple times during the day.

Our respondents generally preferred modern snacks, highlighting the flavour, low price, widespread availability, attractive packaging, and ease to carry and eat while engaging in other activities. We were told that traditional snacks are only sold in local markets or street food stalls, get stale or bad quickly, are messy to eat, and are less tasty. Several high school girls were unfamiliar with or disliked the taste of traditional snacks, with one girl (R2) from Yogyakarta stating,

I prefer modern (snacks) because of the taste, for example, the traditional ones like risoles (fried spring rolls), contain vegetables, but I don't like vegetables, but the modern ones have many flavors.

Another high schooler (R4) from Surabaya said,

It (traditional snacks) tastes like it could make you sick, sometimes there's a weird taste, we are used to eating modern but not traditional food.

Most girls reported that snacking plays an important role in their lives and is habitual and addictive. Motivations for snacking included boredom, waiting time, loneliness, or food cravings, with snacks providing a *teman* (companion) when alone, studying, watching television, or browsing the phone. Fewer respondents mentioned that snacks assuage hunger pangs. This girl (R13) whose work involved managing data in Central Jakarta declared,

During my break, when I am lazy at home, watching TV, or when I travel, I must buy snacks. When I work I snack, so I don't feel bored.

A college respondent from Yogyakarta (R12) stated,

I want to eat, but don't feel like eating something heavy, it means I need to eat snacks. And when I'm studying, because studying can get boring, then I'll buy snacks.

Respondents reported that snacks provide diversity and an opportunity to experiment with new tastes, with popular items such as chips or puffed snacks offered in an assortment of exotic flavours and colours. For instance, one employed respondent indicated that her propensity for puffed snacks is guided by the exciting flavours such as chocolate barbecue, barbecue chicken, salt cheese, spicy barbecue, cheeseburger, seafood, and strawberry. In contrast, girls described home‐made meals as monotonous. A high school respondent (R2) from Yogyakarta indicated,

If we only eat rice, vegetables, it's boring, we need variation in eating, when I don't bring lunch from home, I buy food, a lot of snack foods are cheaper, so I can get full by eating many snacks.

##### Views on health, body size, and nutrition

A healthy girl was described as having a well‐proportioned, strong body, exercising regularly, energetic, free of illness, and maintaining a fresh, cheerful face, with tall girls considered more attractive. Many girls acknowledged the necessity of eating a balanced diet three times daily, limiting *jajan*(ing) and consuming fruits and vegetables for good health. Although most respondents considered themselves healthy, they admitted to not thinking about health. Key informants reported that adolescents believe good health is guaranteed for them regardless of eating habits.

Most high school girls complained of being short, and the majority of respondents were either comfortable with their weight or considered themselves too skinny and were trying to gain weight to improve their body shape. The few who felt they were overweight in the past or present had tried dieting, which typically involved reducing meals, particularly rice intake. An employed girl (R13) from Central Jakarta stated,

Snacks don't make me fat. Like now, no one says I'm getting fatter. It's safe (to eat snacks). People often exaggerate that snacking will make you fat. The heavy foods make you fat, I eat a little rice, but eat snacks often …. Rice has the most significant influence on weight, that's why I reduce (rice) intake now.

Many girl respondents maintained that snacks are not fattening. Key informants confirmed that girls try to lose weight by skipping meals, with mothers sanctioning this as an effective strategy.

Girl respondents typically described nutrition as food ingredients needed to stay healthy and strong, with some referring to nutrients such as carbohydrates, protein, and vitamins. Many girls cited a defunct government slogan, namely, “4 *sehat*, 5 *sampurna* (4 healthy, 5 perfect),” 1The message is that if you consume a balanced diet including four food groups (carbohydrates, protein, vegetables, and fruit), your diet is healthy and if you also drink milk, your diet is perfect. promoting consumption of a balanced diet. Healthy foods were described as home‐made due to safety and cleanliness, whereas unhealthy foods contain monosodium glutamate and preservatives or have extreme colours or flavours, are high in oil and fats, and may be unhygienic, with fast, junk, and fried street foods mentioned as examples. Several expressed other apprehensions about fried street foods, reporting that vendors are notorious for reusing oil or adding plastic to enhance the consistency. Most confessed not paying attention to nutritional content when choosing snacks, admitting they are uninformed and do not consider nutrition important, once again stressing taste the priority. Key informants underlined that girls have limited understanding of nutrition and the risks of consuming snacks high in fat and sugar, with many girls believing snacks have limited calories. One key informant said,

Usually they snack a lot thinking it doesn't have any calories, like kerupuk (traditional fried crackers). No calories. Or fritters. No calorie in it. Sweet drinks, it's only a drink.

#### 3.3.2 Environmental influences

##### Family

Parents were consistently named the most influential people in the girl respondents' lives, guiding major decisions related to school and career, as well as choices concerning extracurricular activities and relationships. High school and employed girls indicated that parents place restrictions on social activities, whereas parents of college students were reported to give more freedom. Girls share personal issues with their mothers, who they frequently called friends.

Particularly during the high school years, mothers were reported to try to influence routine behaviours in the home, including food habits. Mothers of girls living at home typically provide breakfast, a packed lunch, and a dinner meal, which is sometimes purchased or composed of foods quick to prepare and generally served in a central room, with family members free to help themselves and eat in different locations of the house. Many girls indicated that their mothers cook vegetables infrequently, with some explaining that their mothers know they do not like vegetables. Several girls reported fruits to be regularly available at home, although admitted to only eat fruits when pushed by their mothers. Other examples of parental involvement in eating behaviour included insisting that breakfast is consumed, promoting packaged rather than traditional snacks due to perceptions about better hygiene and safety, and discouraging street foods. Key informant mothers' snack food selection criteria focused on food safety such as avoiding flavour enhancers and street vendors.

Most girls reported that their parents, especially mothers, purchase large quantities of snack foods on a routine basis, with some indicating that whatever they request, their mothers buy. Key informant mothers confirmed buying snacks according to their children's desires even if they themselves consider the snacks unhealthy, explaining that mothers cave in to daughters' wishes. One key informant mother regularly replenished separate stores of snacks that catered to each of her children's preferences. Another mother, whose daughter commonly skipped dinner, explained that her daughter places a line of snacks on her desk while studying, stating,

If I give her the option to choose between me or snacks, she may choose snacks. She needs snacks to focus. If she doesn't have snacks, it is difficult for her to study.

Even in the few households where parents did not regularly purchase snacks, girls were permitted to buy and store packaged snacks.

All parents of high school and college students provided a relatively large stipend, ranging from 10,000 to 42,857 Rupiah per day (0.74–3.17 USD), with much of the money intended for food purchases while the girls are outside the household. Working respondents used earned money to purchase snacks.

##### Friends

Our respondents typically had a close set of friends they hang out with during school or work breaks or in eateries, malls, and movie theatres. Girls indicated that friends influence fashion, clothing, and hairstyles. Key informants emphasized that adolescents strive to be accepted by their peers, making alterations in their appearance or behaviours to adhere to social trends.

Eating is a common activity done with friends, often serving as an impetus for frequenting fast food restaurants, cafes, *warungs*, and trendy specialty food shops. Respondents alluded to the social significance of snacking with friends. One high schooler (R3) living in Malang said,

In junior high I *jajan*(ed) less. In high school I see my friends *jajan* a lot, so I jumped on the bandwagon. The way I socialize has changed. In high school, I socialize more often and with more people. I *jajan* more because I have more friends, we go to the cafeteria together, *jajan(ing)* has become a habit.

Even if girls are not hungry, they described pressure to participate in snacking during gatherings, emphasizing the social benefits of snack foods, with snacking reported to strengthen bonds and pleasure with friends. A high schooler (R2) in Yogyakarta stated,

Sometimes it can make us closer, for example when we are chatting, but we are not close yet, I feel it is important to offer snacks to get closer. It would be awkward if I offered rice.

This employed girl (R13) from Central Jakarta said,

When I gather with friends, during *arisan* (social gatherings), when we sing Karaoke, we always buy snacks …. It feels good to talk and snack at the same time.

Our respondents commonly discovered new snack foods through talking to friends or observing what friends eat.

##### Physical

The research areas offered a plethora of modern foods in a range of settings and venues, entailing convenience stores, minimarkets, and supermarkets; shopping malls that typically house a wide range of restaurants including fast food chains, cafes, small food shops, and food courts; and street carts, food stands, and *warungs*. Local markets selling traditional delicacies were less prevalent. Canteens are located in high schools and colleges, with multiple stands offering both meal and snack foods such as packaged cookies or candy, fried foods or bread, or traditional foods such as dumplings during school breaks and lunchtime. Food vendors also position themselves outside schools, selling items popular among teens such as fried foods and fruit juices, which students purchase during breaks or after school.

#### 3.3.3 Macrosystems

The research cities had extensive Internet networks, making Internet both widely available and affordable. Our respondents who all had smartphones consistently cited the Internet as their primary source of information, with social media sites the most frequently visited. Social media was reported to be infiltrated with food advertisements and culinary information, including well‐liked eateries, restaurant promotions, cooking tutorials, and popular snack foods. Information on new and trendy eateries and snacks was also obtained through friends' social media accounts. Many respondents reported watching television routinely, indicating that television commercials frequently promote snack foods. Television was cited as the primary source of information related to food hygiene and safety.

Key informants indicated that social media defines the way adolescents obtain news, providing continually changing and exciting visually dominated information, which sparks their incessant desire to browse popular apps to follow trends and connect with friends. They stressed that the Internet is altering the way adolescents interact and express themselves, facilitating the opportunity to acquire information and communicate rapidly at any time through predominantly visual mediums and making digital connectivity critical to daily activities, including eating.

## 4 DISCUSSION

This qualitative study explored individual, social, physical, and macrosystem factors influencing snacking patterns and behaviours of urban dwelling adolescent girls in Java. The widespread availability of affordable snacks and inclination to consume tasty foods make snacks appealing substitutes for meals. Snacks provide a distraction to boredom, solace when girls are alone, and an enhancement to social gatherings. Girls exhibited limited understanding or concern about the contents or potential negative effects of snacking. Parents facilitated acquisition of unhealthy snacks, whereas friends applied social pressure for routine consumption of snack foods. Extensive networks and use of social media infiltrated with information about eateries and snacks are likely contributing to the already strong yearning for snack foods. The in‐depth nature of the study illuminated details related to individual motivations and environmental factors guiding snacking practices critical to the development of behavioural change strategies.

On an individual level, the findings show a high frequency of snacking primarily driven by the exotic flavours and alluring tastes offered, which when consumed provide instant pleasure. Notably, the most preferred snacks were dominated by modern foods, with findings indicating that consumption of traditional snacks, with the exception of fried foods, is on the decline. Adolescent girls are opting for the cheap, unhealthy packaged snacks saturating Javanese cities, whereas healthy options are less affordable and accessible and difficult to store. On an emotional level, snacking provides an antidote to boredom and loneliness. It is also a habitual accompaniment to routine behaviours such as watching television. A common thread is that snacking is done while girls are alone, often in their bedrooms, taking the form of an additive habit. Eating in solitude and while distracted may facilitate fewer inhibitions and lead to intake of larger quantities of snack foods (Pliner & Bell, 2009). Limited understanding of nutrition together with lack of concern of the potential health ramifications appears to sanction routine snacking in adolescent girls. Research in other contexts has shown that adolescents prioritize immediate gratification provided by taste and popularity over health and nutrition (Cross, Babicz, & Cushman, 1994).

Traditionally, Indonesian mothers are responsible for overseeing the daily activities and needs of their children, including those related to food. In addition to providing three meals daily, most mothers routinely purchase a variety of packaged snacks high in fat and sugar. Adolescent girls' partiality for snacks combined with availability of snack foods at home leads to skipping meals, which are considered heavy and monotonous. Meal skipping was more frequent in respondents living away from home and parental care. The perception that skipping meals is an effective way to lose or maintain weight and that snacks are “light” and low in calories also affects eating patterns, particularly the tendency to replace a rice/noodle dinner—an optional meal not necessarily consumed with family members—with snacks. The evening is also the time when girls are studying or browsing the Internet, making packaged snack foods a convenient food option. Other studies have shown that snacking can lead to meal skipping in adolescents (Savige, Macfarlane, Ball, Worsley, & Crawford, 2007). In other contexts, regular consumption of family meals has been linked to better dietary diversity, nutrient and micronutrient intake and food choices, and may have a positive influence on the quality of diets and meal patterns in young adulthood (Cusatis & Shannon, 1996; Lachat et al., 2012; Larson et al., 2007; Neumark‐Sztainer, Hannan, Story, Croll, & Perry, 2003; Sjoberg, Hallberg, Hoglund, & Hulthen, 2003).

Mothers' roles as confidents and their desire to appease their daughters appear to create an opportunity for girls to dictate the selection of household meals and snack foods and to choose to skip meals. Our respondents reported that home meals often have limited vegetable content, which may be guided by mothers' efforts to align meal ingredients to preferences of their adolescent girls, many of whom reported a dislike for vegetables. Study results also highlight the tendency for mothers to obtain packaged snacks preferred by their children, with findings suggesting that both adolescent girls and mothers are concerned about food safety and cleanliness, which they associate with packaged snacks, but not nutritional content. These factors work synergistically to enable snacking on foods with limited nutrient value and general poor dietary intake. Recent research on child feeding in eastern Java suggests that snacking patterns may start at an early age when mothers engage in “child‐led feeding” involving the provision of foods children like and avoidance of what they dislike, which entails habitually offering packaged snack foods (Global Alliance for Improved Nutrition & Ministry of Health Indonesia, 2013). The same study highlighted mother's lack of understanding of snack food contents and the nutrient needs of young children.

Although parents try to enact restrictions on social activities, when adolescents reach high school age, they are away from home for long stretches of time. This also coincides with the age when adolescents explore self‐identify and autonomy, have a strong attraction to novelty, and experience a heightened desire to be accepted by peers and adhere to social norms. Eating and snacking provides an opportunity for girls to express new aspects of their personality by exerting their own decision making, experimenting with new tastes and environments, and solidifying friendships. Snack foods are perceived to enhance social interactions and bond relations, and our respondents experienced pressure from friends to snack while socializing. Friends were also reported to introduce new snacks and influence snack food choices. Many snack foods are affordable for the pocket money routinely provided by parents, allowing newly empowered girls to make unmonitored purchases of snack foods in or nearby school on a daily basis. Employed girls had more spending autonomy, giving them greater freedom to buy desired food. Snack foods offered in school canteens, street vendors outside schools, convenience stores, or malls were dominated by nutrient‐poor choices, with healthy alternatives such as fruits unavailable. The widespread availability of venues and low price make snacks highly accessible.

Frequent use of the Internet by our respondents, particularly social media, facilitated ongoing exposure to commercial marketing of food promotions and popular eateries and snack foods. Internet use suits the sedentary lifestyles of the girls studied, many of whom reported disliking or finding exercise difficult to do despite the perceived benefits. Internet and social media are a relatively new phenomenon in Indonesia, with extensive networks and affordable prices facilitating widespread use. A recent survey showed that Internet penetration has reached 51.8%, with 75.5% (24.4 million) of Indonesian youths aged 10–24 Internet users (Indonesia Internet Service Provider Association, 2016). Rapid growth of Internet users is causing revolutionary changes in marketing, social interactions, and habitual behaviours, which appears to include those related to food choices and snacking behaviour. Combined with the inundation of the global and national food industry, this is likely intensifying social pressure to consume packaged, prepared, and fast foods, at least in urban adolescent girls.

This study highlights multiple factors at the individual, social, environmental, and macrosystems levels contributing to snacking and the contexts in which adolescent girls snack, illuminating the vulnerability of adolescents to nutritional risks. Results elucidate consistent patterns across urban settings involving frequent snacking, poor food choices, and limited exercise, raising serious concerns about associated health implications and health costs. Snacking involving excessive intake of nutrient‐poor foods high in fats, sugar, and salt combined with little physical activity can contribute to the development of overweight and obesity, which is emerging as a major public health problem in Indonesia, particularly in urban areas and for those with underlying stunting early in life (Ismail, Herini, Hagung, & Sadjimin, 1999; National Institute of Health Research and Development, 2013; Padmiari & Hadi, 2003; Soekirman, Martianto, Atmarita, & Marks, 2003; Usfar, Lebenthal, Achadi, & Hadi, 2010). The fact that girls do not cook, exposure to snack food promotion is increasing, girls are poorly informed and not concerned about good nutrition, and there are no major efforts to combat the snacking trend suggests that these eating patterns will persist, at a time when lifestyle behaviours, including eating, are established. Although the conceptual model used in this qualitative study helped to capture multiple and interacting factors influencing food choices and intake, we omitted to examine certain determinants of eating behaviours, such as household socio‐economics and mother's education, shown to be associated with nutritional status of adolescent girls in Indonesia and elsewhere (Kunto & Bras, 2018; Madjdian et al., 2018). We recognize that a wider range of potential determinants of eating patterns exist, as presented in a recently developed conceptual framework on adolescent malnutrition based on a systematic review of quantitative studies, which can be analysed through quantitative assessments (Madjdian et al., 2018). Our findings underline the urgent need to develop contextually relevant, targeted behavioural change strategies to modify the potentially harmful eating and activity patterns of adolescent girls identified in this study and to curb the trajectory of overweight in urban Indonesia.

## 5 CONCLUSION

In Indonesia, the coexistence of a high prevalence of overweight and undernutrition signals a double burden of malnutrition among adolescents living in urban centres. The pattern of poor eating behaviours and lack of physical activity illuminated in this study, combined with an escalating reliance on social media, underline the urgent need to develop policies and programmes to combat extreme forms of malnutrition simultaneously through targeted approaches. From a sociobehavioural standpoint, the challenges to address the nutritional problems identified are heightened due to the susceptibility of adolescents to environmental influences and unhealthy lifestyles and a tendency to disregard long‐term consequences of potentially harmful behaviours typical of this age group (WHO, 2005). Unless the trend of excessive snacking and meal skipping is addressed, eating behaviours in urban adolescent girls are likely to deteriorate, leading to further rise in overweight and associated chronic diseases, which over the long term, can have devastating effects on the health and economy in Indonesia. The Indonesian Ministry of Health (2015) recognizes the problem and has responded by introducing regulations on processed and fast foods high in sugar, salt, and fat that will take effect in 2019. The research highlights other potential strategies to motivate healthy eating in both early and late adolescence. For instance, schools could be used as a delivery platform to improve dietary intake through behaviour change communication, including peer‐based approaches, and the availability of healthy foods. Policy could be developed to control nutrient‐poor food choices in and around the school setting. Social media campaigns targeting mothers could be designed to encourage the availability of healthy snacks and preparation of healthful foods in the home. Interventions are also essential to ensure that young women in Indonesia, where 14% of adolescents marry before the age of 18 (UNICEF, 2016) and nearly a quarter of women are either mothers or pregnant by the age of 19 (Statistics Indonesia [BPS], National Population and Family Planning Board [BKKBN], Ministry of Health, & ICF International, 2013), enter first pregnancy in a sound nutritional state. Further research is needed to examine dietary patterns in rural adolescents representing different socio‐economic segments of the population and to study eating behaviours during early adolescence.

## CONFLICTS OF INTEREST

The authors declare that they have no conflicts of interest.

## CONTRIBUTIONS

MVL, LSB, EKS, SS, RM, DI, and AT designed the study. INY supervised field operations, and AM conducted data collection. LSB, AM, and INY carried out data analysis and interpretation. This paper was written by LSB with substantial input from all other authors. All authors have reviewed and approved the submitted manuscript.
